# Socioeconomic status and survival of cirrhosis patients: A Danish nationwide cohort study

**DOI:** 10.1186/1471-230X-9-35

**Published:** 2009-05-18

**Authors:** Peter Jepsen, Hendrik Vilstrup, Per Kragh Andersen, Henrik Toft Sørensen

**Affiliations:** 1 Department of Clinical Epidemiology, Aarhus University Hospital, Aarhus, Denmark; 2 Department of Medicine V (Hepatology and Gastroenterology), Aarhus University Hospital, Aarhus, Denmark; 3 Department of Biostatistics, Institute of Public Health, University of Copenhagen, Copenhagen, Denmark

## Abstract

**Background:**

Low socioeconomic status is a risk factor for liver cirrhosis, but it is unknown whether it is a prognostic factor after cirrhosis diagnosis. We examined whether marital status, employment, and personal income were associated with the survival of cirrhosis patients.

**Methods:**

Using registry-data we conducted a population-based cohort study of 1,765 Danish cirrhosis patients diagnosed in 1999–2001 at age 45–59 years. Follow-up ended on 31 December 2003. With Cox regression we examined the associations between marital status (never married, divorced, married), employment (employed, disability pensioner, unemployed), personal income (0–49, 50–99, 100+ percent of the national average) and survival, controlling for gender, age, cirrhosis severity, comorbidity, and substance abuse.

**Results:**

Five-year survival was higher for married patients (48%) than for patients who never married (40%) or were divorced (34%), but after adjustment only divorced patients had poorer survival than married patients (adjusted hazard ratio for divorced vs. married = 1.22, 95% CI 1.04–1.42). Five-year survival was lower for disability pensioners (31%) than for employed (46%) or unemployed patients (48%), also after adjustment (adjusted hazard ratio for disability pensioners vs. employed = 1.35, 95% CI 1.09–1.66). Personal income was not associated with survival.

**Conclusion:**

Marital status and employment were associated with the survival of cirrhosis patients. Specifically, divorced cirrhosis patients and cirrhosis patients who never married had a poorer survival than did married cirrhosis patients, and cirrhosis patients who were disability pensioners had a poorer survival than did employed or unemployed cirrhosis patients. The poorer survival for the divorced and for the disability pensioners could not be explained by differences in other socioeconomic factors, gender, age, cirrhosis severity, substance abuse, or comorbidity. Personal income was not associated with survival.

## Background

Liver cirrhosis is a chronic disease with a median survival time in Denmark of about three years following diagnosis [1]. In the white U.S. population aged 45–54 years cirrhosis was the fifth leading cause of death in 2004, after cancer, heart disease, accidents, and suicide [2]. Studies have found an association between low socioeconomic status and increased cirrhosis incidence [3-6], but it remains unclear whether low socioeconomic status is also associated with a worse prognosis for patients with cirrhosis. There is evidence in favor of such an association from studies of patients with alcoholism [5], cancer [7,8], heart failure [9], stroke [10], or myocardial infarction [11,12], but this topic has not been addressed among cirrhosis patients [13]. Several factors might contribute to a worse prognosis for cirrhosis patients of low socioeconomic status: They may be less prone to seek medical assistance at the first symptoms of cirrhosis; have a greater prevalence of substance abuse or comorbid diseases; receive an inferior treatment; or be less compliant with the given treatment. Accurate information on socioeconomic prognostic factors for cirrhosis patients may improve our understanding of the clinical course of the disease and may also lead to the identification of patients who require special interventions. We therefore examined whether marital status, employment, and personal income were associated with survival of Danish cirrhosis patients.

## Methods

Denmark's 5.3 million inhabitants have access to free tax-supported healthcare, and all acute-care hospitals are public. The unemployment rate is low (around five percent of the workforce aged 45–59 years in 1999–2001 [14]), and economic support is provided to low-income groups. The Danish welfare system aims to reduce socioeconomic inequalities in health [15], but there are no economic benefits associated with the diagnosis of a chronic disease, such as cirrhosis.

According to Danish law, studies that are based exclusively on publicly available data from administrative registries, such as this, require neither ethical approval nor patient consent.

### Data sources

#### Integrated Database for Labor Market Research (IDA)

The IDA database, established in 1990 and administered by the government agency Statistics Denmark, contains socioeconomic information at the individual level for each Danish citizen. The data are primarily supplied by tax authorities, educational institutions and employment services. The IDA database is updated annually on 31 December [16].

#### National Patient Registry

The National Patient Registry contains data from all inpatient admissions to public and private non-psychiatric hospitals in Denmark since 1977 and from outpatient and emergency room visits since 1995 [17]. Each discharge record includes service dates (dates of admission and discharge for inpatients, dates of first and last visit for a given condition for outpatients, and date of visit for emergency room patients), one primary diagnosis and up to twenty secondary diagnoses, and surgical procedures performed. Diagnoses are coded according to the 10^th ^revision of the International Classification of Diseases (ICD-10), but before 1994 they were coded according to the 8^th ^revision (ICD-8). Diagnosis codes are specified by a physician; for inpatients they are given at hospital discharge, for outpatients they are given at the last visit in a series of outpatient visits. Procedure codes are coded according to the Nordic Classification of Surgical Procedures. Unless otherwise noted, we defined diseases using diagnoses from inpatient hospitalizations, outpatient visits, and emergency room visits.

#### Civil Registration System

Denmark's Civil Registration System records dates of birth, death, and emigration for all Danish citizens and is updated daily. This authority also assigns a unique personal identifier to each citizen, and this identifier was used to link individual-level data from the IDA database, the National Patient Registry, and the Civil Registration System [18].

### Information on cirrhosis patients

We identified all patients with a diagnosis of cirrhosis (ICD-8 codes 571.09, 571.92, and 571.99; ICD-10 codes: K70.3 or K74.6) made during an inpatient hospitalization or an outpatient visit between 1 January 1977 and 31 December 2001. Patients eligible for inclusion in this study had to have their first cirrhosis diagnosis recorded during the years 1999 through 2001 and had to be 45–59 years at the time of diagnosis. The restrictions by calendar year and age were applied in order to select patients of working age and to reduce differences in living conditions.

Severity of cirrhosis was based on hospital diagnoses given at the time of inclusion into the study. We identified the presence of variceal bleeding (ICD-10 code I85.0), liver failure (ICD-10 codes K72.x), and bacterial infection (ICD-10 codes K65.x [spontaneous bacterial peritonitis], J13.x-J18.x [pneumonia], N10.x [pyelonephritis], N30.x [cystitis], A46.x [erysipelas], I33.x [endocarditis], and A40.x-A41.x [septicemia]) [19]. We also ascertained whether the patient was an inpatient or outpatient at the time of diagnosis.

#### Socioeconomic status

An individual's socioeconomic status refers to his or her position in society, based on a combination of educational, occupational, and economic criteria [20]. We used information on marital status instead of education because we did not have data on education, and because marital status is one of several factors that are likely to be associated with socioeconomic status [20]. Thus, we obtained information on the patients' marital status on 31 December of the year of cirrhosis diagnosis and on 31 December of each of the five preceding calendar years (never married, divorced/widowed, or married/cohabiting); employment during the majority of the calendar year in which cirrhosis was diagnosed and during each of the five preceding calendar years (employed, disability pensioner, or unemployed); and taxable personal income during the calendar year of cirrhosis diagnosis and during each of the five preceding calendar years (0–49 percent, 50–99 percent, or 100+ percent of the average income in the same calendar year for all Danish citizens of the same gender and age). Information on marital status and personal income in the year of cirrhosis diagnosis was missing for patients who died in that year, so we assumed that they were the same as in the previous year.

#### Substance abuse

For each patient we counted the number of diagnoses of alcohol abuse (ICD-10 codes F10.x [except F10.0 and F10.1], G31.2, K70.x, and K86.0) in the five years before cirrhosis diagnosis (in categories of 0, 1–4, 5–9, or 10+ diagnoses), and we ascertained whether the patient had received a diagnosis of substance abuse other than alcohol abuse during the same period (ICD-10 code F1x.x, except F10.x).

#### Comorbidity

We measured comorbidity using the patients' diagnoses in the five years preceding their cirrhosis diagnosis. The Charlson comorbidity index (in categories of 0, 1, 2, or 3+) served as an overall measure [21], and comorbid diseases were defined according to Quan et al [22]. The index includes mild and severe liver disease, but they were not counted as comorbidities. Additional comorbidity measures were the presence of hospital diagnoses for psychiatric disease (ICD-10 codes Fxx.x, except F1x.x) and the number of inpatient hospitalizations in the five years before the cirrhosis diagnosis (in categories of 0–1, 2–4, 5–9, or 10+).

### Statistical analysis

Patients whose first cirrhosis diagnosis originated from an inpatient hospitalization were followed from the discharge date of that hospitalization, and patients whose first cirrhosis diagnosis originated from an outpatient visit were followed from the last visit in that series of outpatient visits. All patients were followed until death or emigration, or were censored on 31 December 2003, whichever came first. The study outcome was survival time. Patients who died during the hospitalization associated with their first cirrhosis diagnosis were assigned a survival time of 0.5 days. Analyses were based on socioeconomic data for the calendar year preceding the cirrhosis diagnosis, unless otherwise specified.

We computed survival probabilities using the Kaplan-Meier method and used Cox proportional hazards regression to estimate hazard ratios. First, we computed the crude hazard ratios for marital status, employment, and personal income. Second, we included all three markers of socioeconomic status in one Cox model together with gender, age at cirrhosis diagnosis, cirrhosis severity (encompassing variceal bleeding, liver failure, bacterial infection, and inpatient status at time of cirrhosis diagnosis), substance abuse (encompassing number of diagnoses for alcohol abuse and other substance abuse), and comorbidity (encompassing Charlson comorbidity index, psychiatric disease, and number of inpatient hospitalizations). Using Schoenfeld residuals, we determined that hazard ratios were constant over the follow-up time.

We examined whether the timing of the socioeconomic status measurement affected the hazard ratios for the three markers of socioeconomic status. This was done by substituting the marital status, employment, and income data used in the fully adjusted Cox regression model with the same information for the year of cirrhosis diagnosis and for earlier calendar years.

## Results

We included 1,765 cirrhosis patients, of whom 68 percent were men. During a total follow-up time of 3,855 years, 877 patients (50 percent) died; none received a liver transplant. Forty-one percent were married, 40 percent were divorced, less than one-third were employed, two-thirds had a personal income less than fifty percent of the national average, and six percent had an income above the national average (Additional file 1). Eighty-five percent of all cirrhosis patients had one or more diagnoses of alcohol abuse (Additional file 1), and 17 percent were divorced disability pensioners earning less than half the national average.

Compared with married patients, divorced or never-married patients were more likely to be disability pensioners, to have a low income and to abuse alcohol. Employed patients were more likely than the others to be married, have a higher income, and not abuse alcohol, whereas disability pensioners were more likely to be divorced, abuse alcohol, and have comorbidities. Patients in the highest income category were more likely than others to be employed, female, and old. Cirrhosis severity was unrelated to socioeconomic status (Additional file 1).

**Figure 1:**
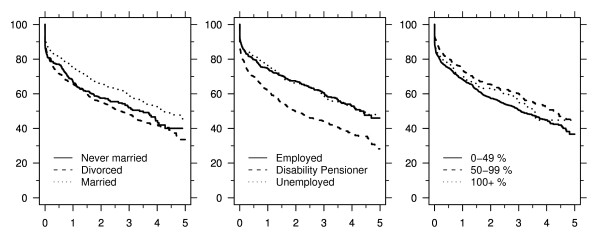
**Survival with respect to time (years) after cirrhosis diagnosis by marital status, employment, and personal income (in percent of the national average income for citizens of same age and gender)**.

Five-year survival was lower for divorced patients (34 percent, 95% CI 28–40) and patients who never married (40 percent, 95% CI 32–48) than for married patients (48 percent, 95% CI 43–53), and it was clearly lower for disability pensioners (31 percent, 95% CI 25–37) than for employed (46 percent, 95% CI 39–52) or unemployed (48 percent, 95% CI 42–54) patients. By contrast, patients earning less than half the national average had only slightly lower five-year survival (38 percent, 95% CI 34–43) than those with higher earnings (45 percent for both categories) (Figure 1). These survival probabilities were consistent with the crude hazard ratios (Additional file 2).

Adjustment for other patient characteristics attenuated the estimate of the prognostic impact of marital status, but the prognosis remained worse for the divorced than for the married (HR = 1.22, 95% CI 1.04–1.42) (Additional file 2). Likewise, the impact of employment was reduced, but being a disability pensioner remained associated with a poorer prognosis (HR for disability pensioner vs. employed = 1.35, 95% CI 1.09–1.66; HR for disability pensioner vs. unemployed = 1.39, 95% CI 1.17–1.65). The prognosis appeared to be worse for those who earned more than the national average, but not by a statistically significant amount (Additional file 2).

The socioeconomic status of the cirrhosis patients deteriorated over the five years preceding their cirrhosis diagnosis. Notably, 27 percent of patients were disability pensioners five years before their cirrhosis diagnosis, and this proportion rose to 39 percent by the end of the year preceding diagnosis. Of the 713 divorced patients, 112 (16 percent) were divorced during the five years preceding their cirrhosis diagnosis. Of the 695 disability pensioners, 577 (83 percent) had not been employed in the five preceding years. This was true of 211 out of a total of 516 unemployed patients (41 percent). Still, substituting socioeconomic information from earlier calendar years or from the year of cirrhosis diagnosis had only a small effect on our findings: the hazard ratios for divorced vs. married patients ranged from 1.21 to 1.30, and those for disability pensioners vs. employed patients ranged from 1.27 to 1.40.

## Discussion

In this nationwide population-based study of 1,765 cirrhosis patients, we found that marital status and employment were associated with survival. Specifically, divorced cirrhosis patients and cirrhosis patients who never married had a poorer survival than did married cirrhosis patients, and cirrhosis patients who were disability pensioners had a poorer survival than did employed or unemployed cirrhosis patients. The poorer survival for the divorced and for the disability pensioners could not be explained by differences in other socioeconomic factors, gender, age, cirrhosis severity, substance abuse, or comorbidity. Personal income was not associated with survival.

The major strengths of our study were access to data from a tax-funded healthcare system with equal access to hospital care and complete follow-up. Use of routinely collected nationwide administrative data on socioeconomic status and hospitalization history ensured that data collection was independent of our study and thus reduced the risk of bias due to differential data validity. At the same time, hospital diagnoses in the National Patient Registry data are not all of high validity [23]. Of particular concern was the validity of cirrhosis diagnoses. A 1985–1990 study of hospital diagnoses of cirrhosis indicated that 15 percent of the diagnoses in the registry did not fulfill diagnostic criteria for cirrhosis [24]. It is possible that these patients, relative to patients who fulfilled the diagnostic criteria, had a better prognosis and a higher socioeconomic status. If so, invalid cirrhosis diagnoses would lead to overestimation of the benefit of high socioeconomic status, but we find it unlikely that such a bias had a substantial effect on our findings.

There are two likely reasons why the socioeconomic status by the end of the calendar year preceding the cirrhosis diagnosis might differ from the status at the time that cirrhosis was established. First, socioeconomic status may change during the time it takes to diagnose the existing cirrhosis. Second, data on socioeconomic status were updated annually and could therefore predate cirrhosis establishment and diagnosis by as much as one year. However, we showed that our findings remained unaffected even after artificially inflating the misclassification of socioeconomic status by increasing the time from measurement of socioeconomic status until diagnosis of cirrhosis. This observation indicates that misclassification of socioeconomic status could not have had a substantial effect on our findings.

The prognostic impact of socioeconomic status among cirrhosis patients has not previously been examined [13], but the survival of Danish cancer patients has been shown to be associated with several markers of socioeconomic status, including marital status, employment, and income [7]. It is not clear why high income was associated with longer survival for Danish cancer patients and not for cirrhosis patients, but in our data the slightly lower survival in the lowest income category could be explained by other patient characteristics; in the cancer study, only gender, age, calendar year, and educational level were considered as possible explanations [7]. The better prognosis for married cirrhosis patients than for divorced cirrhosis patients is also consistent with findings among alcoholic men [5], and among patients with myocardial infarction [11,12].

The mechanisms behind our findings are unclear. A recent study of more than 3,000 patients with myocardial infarction failed to find an association between social support, employment, or income and prognosis, after extensive adjustment for preexisting cardiovascular conditions [25]. This might indicate that we could have explained the prognostic impact of divorce and disability in our study with such patients' alcohol abuse and comorbidity if our data on these characteristics had been sufficiently detailed. However, the unmeasured effects of alcohol abuse and comorbidity would have to be at least as strong as their measured effects to fully explain our findings, and that is unlikely. Additionally, we found that divorced or disabled cirrhosis patients did not have more severe cirrhosis than other patients, which is consistent with a Finnish study of patients hospitalized for alcohol-related disease [26]. Thus, it appears that none of the patient characteristics we considered is capable of fully explaining our findings. We speculate that cirrhosis patients who were divorced or were disabled may have received inferior treatment or were less compliant with the given treatment than were married or disability-free patients. The explanation based on inferior treatment is supported by a Swedish study of women with breast cancer which showed that socioeconomic differences in survival could be partially explained by differences in diagnostic intensity and in appropriate use of radio- and chemotherapy [27]. By contrast, a Finnish study of patients with an alcohol-related disease of the liver or pancreas found no difference in survival according to socioeconomic status, measured by occupation [26]. Unfortunately, we had no data on our patients' treatment or compliance with the treatment, and we did not have data on causes of death, either, but such data might have helped us explain our findings.

Despite the absence of clear mechanisms behind our findings, they might have clinical implications. We showed that divorced or disabled cirrhosis patients had a worse prognosis than did other cirrhosis patients, implying that they may benefit from more frequent follow-up visits in order to ensure compliance with treatment. Additionally, interventions targeting alcohol abuse and comorbidities may reduce the excess mortality for these patients. In addition to standard care, psychosocial therapy may be beneficial, as it has been shown to reduce alcohol dependence and improve social support and quality of life [28,29].

## Conclusion

We found that marital status and employment were associated with survival of cirrhosis patients, whereas personal income was not. Our findings could not be fully explained by differences in other socioeconomic factors, gender, age, cirrhosis severity, substance abuse, or comorbidity.

## Competing interests

The authors declare that they have no competing interests.

## Authors' contributions

PJ and HTS conceived and designed the study. PJ and PKA analyzed the data, and all authors interpreted the data. PJ drafted the manuscript, and HV, PKA, and HTS revised it. All authors read and approved the final manuscript.

## Pre-publication history

The pre-publication history for this paper can be accessed here:

http://www.biomedcentral.com/1471-230X/9/35/prepub

## Supplementary Material

Additional file 1 **Table S1**. Characteristics of the 1,765 cirrhosis patients included in the study. Click here for file

Additional file 2 **Table S2**. The associations of marital status, employment, and personal income with survival for cirrhosis patients. Click here for file
