# Supplementary material for: Socioeconomic status and survival of cirrhosis patients: A Danish nationwide cohort study
Source: BMC Gastroenterol. 2009 May 18;9:35. doi: 10.1186/1471-230X-9-35 (PMC2688507; doi:10.1186/1471-230X-9-35)
Supplement: Additional file 1 — Table S1. Characteristics of the 1,765 cirrhosis patients included in the study. [file 1471-230X-9-35-S1.doc]

| Table 1. Characteristics of the 1,765 cirrhosis patients included in the study. The data on socioeconomic status pertain to the calendar year before cirrhosis diagnosis. The number and percentage of patients in each of the three categories of marital status, employment, and personal income, as well as for the total cohort, are provided in the columns. | | | | | | | | | | | |
| --- | --- | --- | --- | --- | --- | --- | --- | --- | --- | --- | --- |
|  |  | Marital status | | | Employment | | | Personal income | | | Total |
|  |  | Never married | Divorced | Married | Disability pensioner | Unemployed | Employed | 0-49 | 50-99 | 100+ |  |
|  |  | 327 (19) | 713 (40) | 725 (41) | 695 (39) | 516 (29) | 554 (31) | 1180 (67) | 482 (27) | 103 (6) | 1765 (100) |
| Socioeconomic status |  |  |  |  |  |  |  |  |  |  |  |
|  | Marital status |  |  |  |  |  |  |  |  |  |  |
|  | Never married | - | - | - | 156 (22) | 93 (18) | 78 (14) | 254 (22) | 65 (13) | 8 (8) | 327 (19) |
|  | Divorced | - | - | - | 346 (50) | 204 (40) | 163 (29) | 498 (42) | 174 (36) | 41 (40) | 713 (40) |
|  | Married | - | - | - | 193 (28) | 219 (42) | 313 (57) | 428 (36) | 243 (50) | 54 (52) | 725 (41) |
|  | Employment |  |  |  |  |  |  |  |  |  |  |
|  | Disability pensioner | 156 (48) | 346 (49) | 193 (27) | - | - | - | 613 (52) | 72 (15) | 10 (10) | 695 (39) |
|  | Unemployed | 93 (28) | 204 (29) | 219 (30) | - | - | - | 400 (34) | 105 (22) | 11 (11) | 516 (29) |
|  | Employed | 78 (24) | 163 (23) | 313 (43) | - | - | - | 167 (14) | 305 (63) | 82 (80) | 554 (31) |
|  | Income (% of national average) |  |  |  |  |  |  |  |  |  |  |
|  | 0-49 | 254 (78) | 498 (70) | 428 (59) | 613 (88) | 400 (78) | 167 (30) | - | - | - | 1180 (67) |
|  | 50-99 | 65 (20) | 174 (24) | 243 (34) | 72 (10) | 105 (20) | 305 (55) | - | - | - | 482 (27) |
|  | 100+ | 8 (2) | 41 (6) | 54 (7) | 10 (1) | 11 (2) | 82 (15) | - | - | - | 103 (6) |
| Demographics |  |  |  |  |  |  |  |  |  |  |  |
|  | Men | 274 (84) | 459 (64) | 467 (64) | 457 (66) | 337 (65) | 406 (73) | 889 (74) | 262 (54) | 49 (48) | 1200 (68) |
|  | Age at diagnosis |  |  |  |  |  |  |  |  |  |  |
|  | 45-49 years | 158 (48) | 193 (27) | 159 (22) | 197 (28) | 162 (31) | 151 (27) | 360 (31) | 128 (27) | 22 (21) | 510 (29) |
|  | 50-54 years | 101 (31) | 239 (34) | 266 (37) | 239 (34) | 164 (32) | 203 (37) | 408 (35) | 158 (33) | 40 (39) | 606 (34) |
|  | 55-59 years | 68 (21) | 281 (39) | 300 (41) | 259 (37) | 190 (37) | 200 (36) | 412 (35) | 196 (41) | 41 (80) | 649 (37) |
| Cirrhosis severity |  |  |  |  |  |  |  |  |  |  |  |
|  | Variceal bleeding | 24 (7) | 35 (5) | 54 (7) | 32 (5) | 30 (6) | 51 (9) | 73 (6) | 33 (7) | 7 (7) | 113 (6) |
|  | Liver failure | 12 (4) | 30 (4) | 17 (2) | 26 (4) | 14 (3) | 19 (3) | 40 (3) | 15 (3) | 4 (4) | 59 (3) |
|  | Bacterial infection | 29 (9) | 54 (8) | 54 (7) | 61 (9) | 44 (9) | 32 (6) | 101 (9) | 30 (6) | 6 (6) | 137 (8) |
|  | Inpatient at cirrhosis diagnosis | 278 (85) | 563 (79) | 551 (76) | 579 (83) | 411 (80) | 402 (73) | 951 (81) | 368 (76) | 73 (71) | 1392 (79) |
| Substance abuse |  |  |  |  |  |  |  |  |  |  |  |
|  | Alcohol diagnoses |  |  |  |  |  |  |  |  |  |  |
|  | 10+ | 34 (10) | 104 (15) | 41 (6) | 120 (17) | 37 (7) | 22 (4) | 132 (11) | 36 (7) | 11 (11) | 69 (4) |
|  | 5-9 | 52 (16) | 125 (18) | 97 (13) | 127 (18) | 94 (18) | 53 (10) | 199 (17) | 66 (14) | 9 (9) | 174 (10) |
|  | 1-4 | 201 (61) | 402 (56) | 445 (61) | 364 (52) | 324 (63) | 360 (65) | 690 (58) | 297 (62) | 61 (59) | 1258 (71) |
|  | 0 | 40 (12) | 82 (12) | 142 (20) | 84 (12) | 61 (12) | 119 (21) | 159 (13) | 83 (17) | 22 (21) | 264 (15) |
|  | Other substance abuse | 6 (2) | 8 (1) | 6 (1) | 17 (2) | 2 (0.4) | 1 (0.2) | 18 (2) | 1 (0.2) | 1 (1) | 20 (1) |
| Comorbidity |  |  |  |  |  |  |  |  |  |  |  |
|  | Charlson comorbidity index |  |  |  |  |  |  |  |  |  |  |
|  | 3+ | 14 (4) | 29 (4) | 25 (3) | 42 (6) | 11 (2) | 15 (3) | 49 (4) | 16 (3) | 3 (3) | 68 (4) |
|  | 2 | 29 (9) | 58 (8) | 62 (9) | 79 (11) | 34 (7) | 36 (7) | 103 (9) | 38 (8) | 8 (8) | 149 (8) |
|  | 1 | 75 (23) | 160 (22) | 145 (20) | 177 (25) | 95 (18) | 108 (19) | 269 (23) | 94 (20) | 17 (17) | 380 (22) |
|  | 0 | 209 (64) | 466 (65) | 493 (68) | 397 (57) | 376 (73) | 395 (71) | 759 (64) | 334 (69) | 75 (73) | 1168 (66) |
|  | Psychiatric disease | 17 (5) | 48 (7) | 25 (3) | 63 (9) | 18 (3) | 9 (2) | 69 (6) | 18 (4) | 3 (3) | 90 (5) |
|  | Hospitalizations in last five years |  |  |  |  |  |  |  |  |  |  |
|  | 10+ | 16 (5) | 52 (7) | 26 (4) | 67 (10) | 14 (3) | 13 (2) | 70 (6) | 22 (5) | 2 (2) | 94 (5) |
|  | 5-9 | 53 (16) | 123 (17) | 109 (15) | 158 (23) | 72 (14) | 55 (10) | 204 (17) | 64 (13) | 17 (17) | 285 (16) |
|  | 2-4 | 140 (43) | 305 (43) | 289 (40) | 295 (42) | 219 (42) | 220 (40) | 494 (42) | 204 (42) | 36 (35) | 734 (42) |
|  | 0-1 | 118 (36) | 233 (33) | 301 (42) | 175 (25) | 211 (41) | 266 (48) | 412 (35) | 192 (40) | 48 (47) | 652 (37) |
