# Supplementary material for: Socioeconomic status and survival of cirrhosis patients: A Danish nationwide cohort study
Source: BMC Gastroenterol. 2009 May 18;9:35. doi: 10.1186/1471-230X-9-35 (PMC2688507; doi:10.1186/1471-230X-9-35)
Supplement: Additional file 2 — Table S2. The associations of marital status, employment, and personal income with survival for cirrhosis patients. [file 1471-230X-9-35-S2.doc]

| Table 2. The associations of marital status, employment, and personal income with survival for cirrhosis patients. Associations are expressed as hazard ratios (HR), with associated 95% confidence intervals (CI), before (crude HR) and after adjustment for the other variables in the table. | | | |
| --- | --- | --- | --- |
|  |  | Crude HR (95% CI) | Adjusted HR (95% CI) |
| Socioeconomic status |  |  |  |
|  | Marital status |  |  |
|  | Never married | 1.26 (1.05-1.52) | 1.05 (0.86-1.28) |
|  | Divorced | 1.40 (1.21-1.62) | 1.22 (1.04-1.42) |
|  | Married | 1.00 [reference] | 1.00 [reference] |
|  | Employment |  |  |
|  | Disability pensioner | 1.60 (1.37-1.88) | 1.35 (1.09-1.66) |
|  | Unemployed | 1.00 (0.83-1.20) | 0.97 (0.79-1.20) |
|  | Employed | 1.00 [reference] | 1.00 [reference] |
|  | Income (% of national average) |  |  |
|  | 0-49 | 1.12 (0.84-1.50) | 0.80 (0.58-1.11) |
|  | 50-99 | 0.88 (0.65-1.20) | 0.80 (0.59-1.10) |
|  | 100+ | 1.00 [reference] | 1.00 [reference] |
| Demographics |  |  |  |
|  | Male gender | 1.27 (1.09-1.47) | 1.26 (1.08-1.48) |
|  | Age at diagnosis |  |  |
|  | 45-49 years | 1.00 [reference] | 1.00 [reference] |
|  | 50-54 years | 0.94 (0.80-1.12) | 0.93 (0.78-1.10) |
|  | 55-59 years | 1.08 (0.91-1.27) | 1.06 (0.89-1.25) |
| Cirrhosis severity |  |  |  |
|  | Variceal bleeding | 1.22 (0.94-1.58) | 1.06 (0.81-1.39) |
|  | Liver failure | 3.30 (2.47-4.40) | 2.92 (2.17-3.94) |
|  | Bacterial infection | 1.52 (1.21-1.91) | 1.22 (0.96-1.54) |
|  | Inpatient at cirrhosis diagnosis | 1.77 (1.48-2.12) | 1.54 (1.28-1.86) |
| Substance abuse |  |  |  |
|  | Alcohol diagnoses |  |  |
|  | 10+ | 1.96 (1.51-2.55) | 1.37 (0.92-2.04) |
|  | 5-9 | 1.52 (1.19-1.94) | 1.50 (1.14-1.98) |
|  | 1-4 | 1.23 (1.00-1.51) | 1.14 (0.93-1.40) |
|  | 0 | 1.00 [reference] | 1.00 [reference] |
|  | Other substance abuse | 1.70 (1.00-2.88) | 1.03 (0.59-1.80) |
| Comorbidity |  |  |  |
|  | Charlson comorbidity index |  |  |
|  | 3+ | 2.58 (1.95-3.42) | 2.00 (1.49-2.69) |
|  | 2 | 1.76 (1.41-2.19) | 1.52 (1.21-1.91) |
|  | 1 | 1.15 (0.98-1.36) | 1.02 (0.86-1.21) |
|  | 0 | 1.00 [reference] | 1.00 [reference] |
|  | Psychiatric disease | 1.47 (1.12-1.93) | 0.94 (0.69-1.26) |
|  | Hospitalizations in last five years |  |  |
|  | 10+ | 2.37 (1.81-3.10) | 1.46 (1.05-2.03) |
|  | 5-9 | 1.62 (1.34-1.98) | 1.10 (0.88-1.38) |
|  | 2-4 | 1.35 (1.16-1.58) | 1.14 (0.97-1.34) |
|  | 0-1 | 1.00 [reference] | 1.00 [reference] |
